# 气管多形性腺瘤1例

**DOI:** 10.3779/j.issn.1009-3419.2011.03.19

**Published:** 2011-03-20

**Authors:** 亮 郭, 银萍 王, 洪喜 马

**Affiliations:** 130021 长春，吉林大学第一医院病理科 Department of Pathology, the First Hospital of Jilin University, Changchun 130021, China

**Keywords:** 肺肿瘤, 多形性腺瘤, 气管, Lung neoplasms, Pleomorphic adenoma, Trachea

## 1 病例资料

患者女，2 3岁。因“间断咳血一月余”来我院就诊。一月前因“感冒”后出现咳鲜血（约10 mL）伴发热、咳嗽及咳痰，CT扫描示气管肿物，给予抗炎对症治疗后咳嗽、咳痰症状消失。由外院支气管镜活检，病理诊断为良性肿瘤，于2009年1月5日转入我院进一步诊治。支气管镜检查：主气道距隆突近3 cm处见一肿物突向管腔，管腔明显狭窄。CT扫描见气管下段管腔内一类圆形团块影，边缘较光整，基底部与气管左后壁似有蒂相连。手术袖状切除部分气管及肿物后行气管成形术。

病理检查：送检一段气管，长1.8 cm，直径1.6 cm，距上端切缘0.4 cm处见一肿物，体积2.0 cm×1.2 cm× 1.0 cm，肿瘤位于气管粘膜下，未见明显包膜，表面光滑，切面呈实性，灰白色，镜下见上皮细胞分布在粘液样基质中，上皮细胞呈片状及腺样排列（图 1）。免疫组化显示Ki-67阳性率 < 5%，瘤细胞SMA及p63呈阳性表达（图 2）。

**1:**
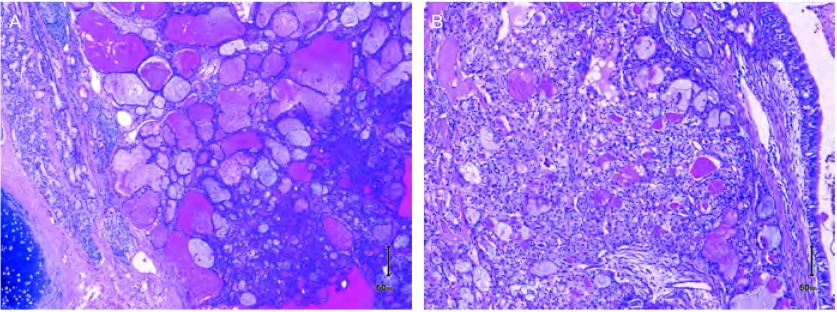
气管多形性腺瘤的HE染色结果。A：肿物位于气管软骨旁，上皮样细胞在粘液样基质内呈腺样及片状排列（HE, ×40）；B：肿物位于气管粘膜下，上皮样细胞在粘液样基质内成片排列（HE, ×100）。 HE stain results of pleomorphic adenoma of trachea. A: The tumor located by bronchial cartilage, glandular and sheets of epitheliod cells scattered in myxoid stroma (HE, ×40); B: Beside the respiratory mucosa sheets of epitheliod cells scattered in the myxoid stroma (HE, ×100).

**2:**
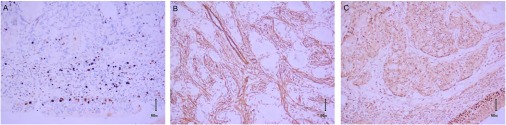
气管多形性腺瘤的免疫组化染色结果。A：Ki-67阳性率 < 5%（IHC, ×200）；B：上皮样细胞中SMA呈阳性（IHC, ×200）；C：上皮样细胞中p63呈阳性（IHC, ×200）。 Immunohistochemical stain results of pleomorphic adenoma of trachea. A: Positive rate of Ki-67 < 5% (IHC, ×100); B. Expression of SMA in epitheliod cells was positive (IHC, ×100); C. Expression of p63 in epitheliod cells was positive (IHC, ×200).

病理诊断：气管多形性腺瘤。

## 2 讨论

多形性腺瘤多发生于涎腺组织，发生于气管者罕见，文献^[1]^报道50余例。此瘤可发生于气管内，也可见于肺实质内。肿瘤直径大小为2 cm-16 cm，多呈息肉样。气管内的病变常累及主支气管或二级支气管，因此表现出阻塞的症状如咳嗽、咳痰、呼吸困难及喘息等。本例患者病变位于气管内，以咳血为首发症状，可能是由于肿瘤侵及血管，导致出血。患者伴有咳嗽、咳痰和感染，与文献^[1]^描述一致。位于肺实质内的病变通常不累及气道，因此多无临床症状，常在查体行影像学检查时被发现^[2]^。

病理检查：气管多形性腺瘤的组织学改变与发生于唾液腺者相同，具有双向分化特征。片状、小梁状或岛状的上皮细胞和（或）肌上皮细胞分布在粘液样、透明软骨样基质或透明变性间质中。可以伴有灶性鳞状上皮化生和纤维化，间质中也可见脂肪组织及脂肪母细胞^[3, 4]^。出现坏死和病理核分裂常提示有恶变的可能，但很少见。免疫组化显示上皮成分CK阳性，肌上皮细胞vimentin、actin及S-100阳性^[5]^。本病例切片中，上皮样细胞中p63和SMA表达阳性，提示该病例以肌上皮样细胞分化为主。同时Ki-67阳性率 < 5%，显示较低的增殖活性。

多形性腺瘤的发生机制尚不清楚^[5]^。诊断时需与类癌、腺样囊性癌、低恶度的粘液表皮样癌、软骨瘤及错构瘤等相鉴别^[6]^。

肿瘤切除和气管重建是目前治疗此瘤的主要方法。少数病例术后有转移或复发的报道，故认为生物学行为具有低度恶性潜能^[4]^。肿瘤的复发取决于肿瘤的大小、局部的浸润程度以及核分裂^[7]^。因此多数学者倾向手术治疗，以防复发。
